# In Vitro Assessment of the Antidiabetic and Anti-Inflammatory Potential of *Artemisia absinthium, Artemisia vulgaris* and *Trigonella foenum-graecum* Extracts Processed Using Membrane Technologies

**DOI:** 10.3390/molecules28207156

**Published:** 2023-10-18

**Authors:** Elena Neagu, Gabriela Paun, Camelia Albu, Oana Teodora Apreutesei, Gabriel Lucian Radu

**Affiliations:** 1 National Institute of Research and Development for Biological Sciences, Centre of Bioanalysis, 296 Splaiul Independentei, 060031 Bucharest, Romania; elena.neagu@incdsb.ro (E.N.); gabriela.paun@incdsb.ro (G.P.); camelia.albu@incdsb.ro (C.A.); 2 Commercial Society for Medicinal Plant Research and Processing Plantavorel, 46 Cuza Voda Street, 610019 Piatra Neamt, Romania; oana.ciuperca@plantaborel.ro

**Keywords:** antidiabetic, anti-inflammatory activity, *Artemisia absinthium*, *Artemisia vulgaris*, *Trigonella foenum-graecum*, membrane technologies, α-amylase, α-glucosidase, hyaluronidase, lipoxygenase

## Abstract

Recently, there has been increased interest in the discovery of new natural herbal remedies for treating diabetes and inflammatory diseases. In this context, this work analyzed the antidiabetic and anti-inflammatory potential of *Artemisia absinthium*, *Artemisia vulgaris* and *Trigonella foenum-graecum* herbs, which have been studied less from this point of view. Therefore, extracts were prepared and processed using membrane technologies, micro- and ultrafiltration, to concentrate the biologically active principles. The polyphenol and flavone contents in the extracts were analyzed. The qualitative analysis of the polyphenolic compounds was performed via HPLC, identifying chlorogenic acid, rosmarinic acid and rutin in *A. absinthium*; chlorogenic acid, luteolin and rutin in *A. vulgaris*; and genistin in *T. foenum-graecum*. The antidiabetic activity of the extracts was analyzed by testing their ability to inhibit α-amylase and α-glucosidase, and the anti-inflammatory activity was analyzed by testing their ability to inhibit hyaluronidase and lipoxygenase. Thus, the concentrated extracts of *T. foenum-graecum* showed high inhibitory activity on a-amylase—IC_50_ = 3.22 ± 0.3 μg/mL—(compared with acarbose—IC_50_ = 3.5 ± 0.18 μg/mL) and high inhibitory activity on LOX—IC_50_ = 19.69 ± 0.52 μg/mL (compared with all standards used). The concentrated extract of *A. vulgaris* showed increased α-amylase inhibition activity—IC_50_ = 8.57 ± 2.31 μg/mL—compared to acarbose IC_50_ = 3.5 ± 0.18 μg/mL. The concentrated extract of *A. absinthium* showed pronounced LOX inhibition activity—IC_50_ = 19.71 ± 0.79 μg/mL—compared to ibuprofen—IC_50_ = 20.19 ± 1.25 μg/mL.

## 1. Introduction

Medicinal plants that have been known and used for centuries remain potential sources for finding new remedies to treat or alleviate various diseases, including diabetes and inflammatory diseases.

Diabetes is a metabolic disorder that leads to severe functional disorders such as neuropathy, vasculopathy, dyslipidemia, retinopathy, and cardiovascular disease [1]. The incidence of this disease is constantly increasing; from 400 million people in 2016, it is estimated that there will be 642 million people with type II diabetes in 2040 [2]. Antidiabetic drugs such as acarbose, miglitol and voglibose reduce the degradation and absorption of sugars by inhibiting the digestive enzymes involved in these processes—α-amylase and α-glucosidase. However, these drugs also have side effects, so alternatives from natural sources are being sought [3].

Drugs used to treat inflammation and pain are nonsteroidal drugs, which generally have numerous side effects such as ulcers and hemorrhages [4,5]. Their long-term use even leads to cardiovascular, metabolic, endocrine and ophthalmologic damage [5].

Medicinal plants are used in folk medicine to treat many inflammatory diseases and continue to be important sources of new anti-inflammatory and antioxidant agents [6,7]. The inhibition of enzymes involved in the inflammatory process such as lipoxygenase and hyaluronidase is the basis for finding new treatments for allergies and inflammatory diseases [8,9]. Oxidative stress generated by reactive oxygen species (ROS) is associated with pathological inflammatory processes and DNA distortions, leading to carcinogenesis and tumors [10]. 

Lipoxygenase (LOX) is involved in inflammatory diseases such as rheumatoid arthritis, inflammatory bowel disease, psoriasis, allergic rhinitis, atherosclerosis and certain types of cancer. During the activity of LOX, peroxy radicals are formed in the reaction medium, which can serve as a source of free radicals. Therefore, antioxidants that scavenge free radicals may also act as LOX inhibitors [11].

Hyaluronidase acts on hyaluronic acid, which is an important component of the extracellular matrix and is involved in many physiological processes such as embryogenesis, wound healing and cell migration [7,12]. Studies have led to the discovery of hyaluronidase inhibitors (tannins, cucumbers, flavonoids) in plants that can be used as anti-inflammatory, anti-allergic and antitumor agents in the treatment of bacterial infections, and as adjuvants in the treatment of arthritis [13,14].

Membrane technologies—micro- and ultrafiltration—are low-cost and effective alternatives to traditional technologies for concentrating biologically active compounds. These processes are characterized by low operating and maintenance costs, ease of operation at moderate temperatures and pressures, high permittivity, and selective separations. [15,16]. These technologies can be successfully used for the purification and concentration of plant extracts, preserving their functional and nutritional properties [17,18].

*Artemisia vulgaris* and *Artemisia absinthium* are two plants of the Asteraceae family (Compositae) used in traditional medicine for their numerous therapeutic properties. 

*Artemisia vulgaris* (known as mugwort) is a temperate shrub of Europe, Asia, North Africa, and North America [19]. The plant is used in folk medicine to treat gastrointestinal and gynecological disorders, to relieve hypertension and nervous system disorders [20,21] and for culinary purposes. Studies have revealed various pharmacological properties of *A. vulgaris*, such as anti-inflammatory, antioxidant, antitumor, and immunomodulatory activities [22,23].

*Artemisia absinthium* L.—also known as wormwood—has been known for centuries and is used in folk medicine for gastrointestinal and urinary tract disorders, fever and helminthiasis [24,25]. *A. absinthium* has been shown to be effective as an antiparasitic and digestive agent [25], and in recent years, it has also been shown to alleviate the symptoms of leukemia, sclerosis, diabetes, malaria and even some types of cancer [26].

*Trigonella foenum-graecum* (also known as fenugreek) is an annual herb in the Fabaceae family that has been known since ancient times for its medicinal and culinary properties. Fenugreek is cultivated and used as a spice in many countries in Asia, Europe and Africa and has high nutraceutical value [27]. In folk medicine, this plant is used primarily to stimulate the immune system and for digestive and reproductive disorders [28]. The seed powder lowers blood sugar and improves symptoms in patients with type 2 diabetes [29]. The seeds and leaves of this herb are rich in flavonoids, alkaloids and saponins, which give it medicinal properties [30]. While the seeds of fenugreek have been extensively studied for treating inflammation, cancer and diabetes, little is known about the leaves.

In the present work, the antidiabetic and anti-inflammatory activity of the following plants was analyzed: *Artemisia vulgaris*, *A. absinthium*, and *Trigonella foenum-graecum*. This was achieved by testing their ability to inhibit α-amylase, α-glucosidase activity, hyaluronidase (HYA) and lipoxygenase (LOX) activity, respectively. The manuscript is original, and the data presented have not been published in another article.

## 2. Results and Discussion

### 2.1. Phytochemical Analysis and Antioxidant Capacity

**Figure 1:**
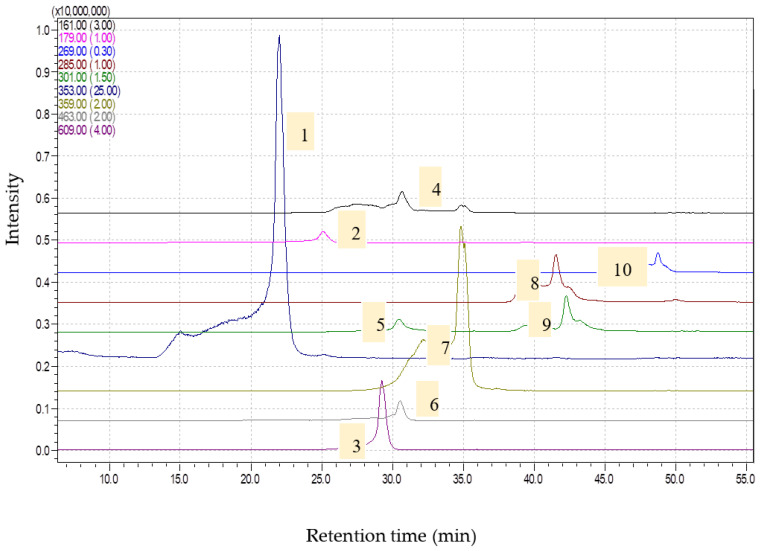
Chromatograms obtained for *Artemisia absinthium* extract (1—chlorogenic acid, [M-H]-353; 2—caffeic acid, [M-H]-179; 3—rutin, [M-H]-609; 4—umbelifferone, [M-H]-161; 5—ellagic acid, [M-H]-301; 6—isoquercitrin, [M-H]-463; 7—rosmarinic acid, [M-H]-359; 8—luteolin, [M-H]-285; 9—quercetin, [M-H]-301; 10—apigenin, [M-H]-269) via HPLC-MS.

**Figure 2:**
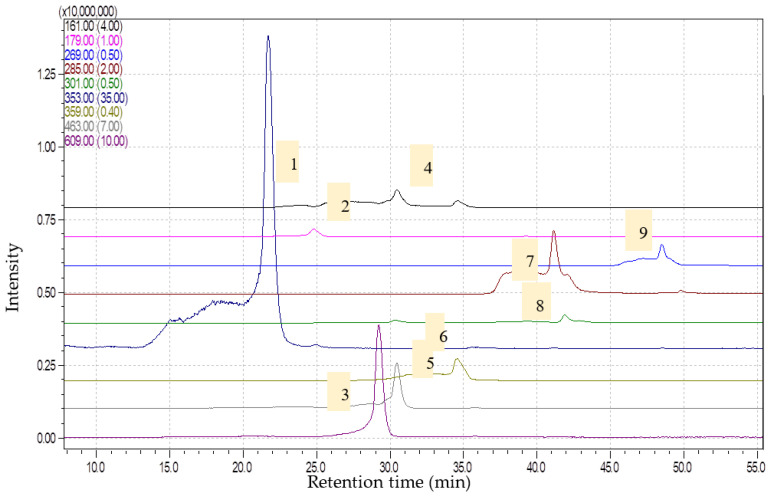
Chromatograms obtained for *Artemisia vulgaris* extract (1—chlorogenic acid, [M-H]-353; 2—caffeic acid, [M-H]-179; 3—rutin, [M-H]-609; 4—umbelifferone, [M-H]-161; 5—isoquercitrin, [M-H]-463; 6—rosmarinic acid, [M-H]-359; 7—luteolin, [M-H]-285; 8—quercetin, [M-H]-301; 9—apigenin, [M-H]-269) via HPLC-MS.

**Figure 3:**
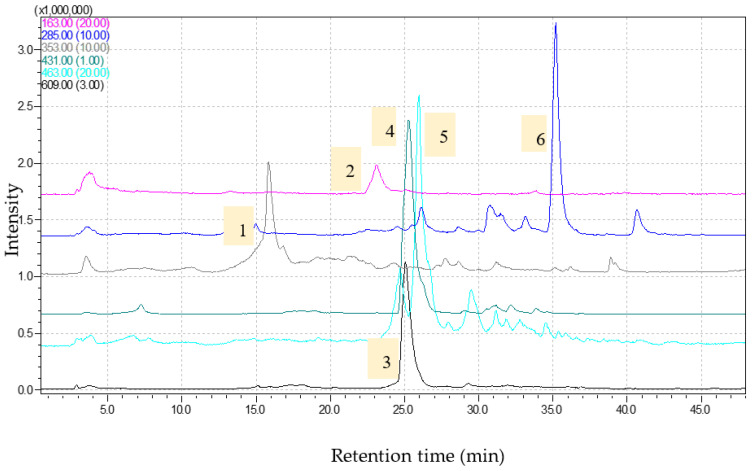
Chromatograms obtained for *Trigonella foenum-graecum* extract (1—chlorogenic acid, [M-H]-353; 2—coumaric acid, [M-H]-163; 3—rutin, [M-H]-609; 4—genistin, [M-H]-431; 5—isoquercitrin, [M-H]-463; 6—luteolin, [M-H]-285) via HPLC-M.

The total polyphenol and flavone contents in the extracts of *Artemisia absinthium, Artemisia vulgaris* and *Trigonella foenum-graecum* and the antioxidant activities of the extracts were analyzed using the spectrophotometric and chromatographic methods presented in the previous chapter, and the results are presented in Table 1, Table 2 and Table 3 and Figure 1, Figure 2 and Figure 3. A significant increase in the content of the analyzed compounds as well as the antioxidant activity of the concentrated extracts compared to the initial (microfiltered) extracts was observed, which illustrates the efficiency of the ultrafiltration process.

Extracts of *Artemisia vulgaris* have been shown to be richer in polyphenolic compounds, with levels almost twice those of *Artemisia absinthium*. A significant increase in the amount of active compounds was obtained in concentrated extracts, especially in the *Artemisia vulgaris* concentrate, from 7877.50 ± 260.96 μg CA/mL to 11,440.21 ± 49.96 μg CA/mL). The amount of flavones was also higher in the extracts of *Artemisia vulgaris* than in *Artemisia absinthium*, with ultrafiltration even doubling the amount of flavones, from 505.18 ± 30.12 μg QE/mL initially to 1020.12 ± 45.12 μg QE/mL in the concentrate, illustrating the efficiency of the ultrafiltration process. The analysis of the *Trigonella foenum-graecum* extract via the HPLC–MS technique revealed the presence of polyphenolic compounds and showed a high amount of genistin.

The results concerning the antioxidant activity of the extracted and processed *Artemisia* extracts are shown in Table 1. It is unanimously agreed that antioxidant activity must be checked using several alternative methods, because the use of only one method is not informative [31,32]. Similar results were obtained with all three methods used; the extracts of *T. foenum-graecum* showed the highest antioxidant activity, followed by *A. vulgaris* and *A. absinthium*, and the antiradical capacity of the concentrated extracts was higher than that of the microfiltrate, illustrating the efficiency of the ultrafiltration process. Data from the literature showed that *T. foenum-graecum* seed extracts were good antioxidant agents, which was attributed to the total phenolic compound content [22,32].

There is a direct correlation between the content of polyphenols and flavones and antioxidant activity, which is consistent with other studies [33,34]. The high content of polyphenols could explain the high antioxidant activity of *Artemisia* extracts [35]. Flavonoids also have antioxidant properties, as shown in other studies on other *Artemisia* species [36]. In the case of the *Trigonella foenum-graecum* extracts, HPLC analysis showed the presence of a significant amount of genistin, which could explain the high antioxidant capacity.

Antioxidant activity is mainly attributed to phenolic compounds because of their ability to protect living systems from oxidative damage caused by free radicals [37]. Scientific studies have shown that antioxidants can prevent serious diseases such as cardiovascular diseases due to their antiradical capacity, and they also have anti-inflammatory and anti-carcinogenic effects [38].

Previous studies have shown that *Artemisia vulgaris* has increased antiradical activity compared to other species of the *Artemisia* genus [39] and that leaf extracts can be used as an effective antioxidant [40]. Regarding *Artemisia absinthium*, some studies have shown moderate antiradical activity [41], while others have shown high antiradical activity correlating with large amounts of polyphenols and flavonoids [42].

Quantitative analysis revealed increased chlorogenic acid content in the two *Artemisia* species, which was significantly higher in *Artemisia vulgaris*. Higher levels of luteolin and isoquercitrin were also found in *Artemisia vulgaris*. The results are consistent with other data in the literature [39].

Rutin was determined in large amounts in both plants. Rutin has a high antioxidant capacity and is reported to be used in the treatment of various diseases related to metabolic syndrome, including diabetes, and neurodegenerative diseases associated with oxidative stress [43]. Numerous scientific reports have claimed that the regular consumption of flavonoids or polyphenols reduces the risk of cardiovascular disease, diabetes and cancer [44]. Rosmarinic acid was detected in a greater quantity in *Artemisia absinthium*. In most extracts, the concentration of active compounds was observed, showing the efficiency of the ultrafiltration process.

### 2.2. α-Amylase and α-Glucosidase Inhibition Activity

Various pharmacological approaches are used to control diabetes. One of them refers to the control of postprandial hyperglycemia by inhibiting glucose uptake in the intestine [45]. This is achieved by taking oral agents that interfere with glucose absorption, i.e., inhibitors of pancreatic a-glucosidase and a-amylase.

The plants used in this study showed high inhibitory activity on α-amylase, especially the concentrated extract of *T. foenum-graecum* (IC_50_ = 3.22 ± 0.30 μg/mL), which was higher than acarbose used as a standard, followed by the concentrated extract of *A. vulgaris* (IC_50_ = 8.57 ± 2.31 μg/mL). The inhibitory activity levels of the concentrated extracts were significantly higher than those of the microfiltrates, which underlines the efficiency of the ultrafiltration process.

*T. foenum-graecum* also showed high inhibitory activity on a-glucosidase (IC_50_ = 11.14 ± 0.90 μg/mL), and the concentrated extract of *A. absinthium* showed moderate inhibitory activity on a-glucosidase (IC_50_ = 31.90 ± 1.89 μg/mL). The concentrated extracts showed higher inhibitory activity than the microfiltrate. The results are shown in Table 3.

Other plants of the *Artemisia* genus, such as *Artemisia campestris* and *Artemisia herba-alba* Asso, are used in traditional medicine to treat diabetes in countries such as Algeria, Morocco, Pakistan and Mexico. The blood glucose-lowering effect of extracts of *Artemisia absinthium* by inhibiting α-glucosidase has been confirmed in other studies [46].

In vitro studies have reported the inhibitory effect of α-amylase activity via a 70% ethanolic extract of *Artemisia herba-alba*. This resulted in an 11% decrease in α-amylase activity, which may be a mechanism by which this extract can lower blood glucose [47]. Studies conducted with another species of *Artemisia—Artemisia indica*—have shown that the antihyperglycemic and antihyperlipidemic effects of some methanolic and chloroform extracts are comparable to those of glibenclamide [48]. Antidiabetic activity has also been demonstrated in other species of the *Artemisia* genus such as *A. amygdalina* [49] and *A. pallens* [50].

The inhibitory effect of *Trigonella foenum-graecum* extracts on α-amylase and α-glucosidase has hardly been reported in the literature. The results obtained are in agreement with previous reports demonstrating the inhibitory effect of *T. foenum-graecum* on amylase activity [51].

### 2.3. Lipoxygenase (LOX) and Hyaluronidase (HYA) Inhibition Activity

Synthetic drugs—non-steroidal anti-inflammatory drugs and anti-rheumatic drugs—reduce the inflammatory process but may also have several side effects. Herbal therapies may be beneficial because they contain several medically important chemical constituents, are readily available, have low costs and have negligible side effects [52].

In the inflammatory process and pathological conditions in general, enzymes such as lipoxygenase (LOX), cyclooxygenase (COX) and hyaluronidase (HYA) are activated, leading to the synthesis of prostaglandins involved in the inflammatory process and allergic diseases [11].

Studies have shown that plant-derived bioactive compounds such as phenolic acids and flavonoids with antioxidant activity also possess anti-hyaluronidase activities, with positive correlations demonstrated between the amount of these compounds and anti-hyaluronidase activity [7].

The extracts tested in this study showed accentuated anti-inflammatory activity by inhibiting LOX: the concentrated extracts of *T. foenum-graecum* and *A. absinthium* showed similar inhibitory activities, IC_50_ = 19.69 μg/mL and IC_50_ = 19.71 μg/mL, respectively, which were even higher than the polyphenolic compound used as a reference standard (i.e., rutin—IC_50_ = 22.34 μg/mL) (Table 4). The concentrated extracts of *Artemisia vulgaris* and *T. foenum-graecum* showed moderate HYA inhibition activity: IC_50_ = 17.18 μg/mL IC_50_ = 17.57 μg/mL compared to ibuprofen, the reference standard used—IC_50_ = 5.73 μg/mL.

The anti-inflammatory effect of *Artemisia vulgaris* extracts is attributed to the presence of flavonoids [53]. Previous studies have shown that extracts of *Artemisia vulgaris* have an anti-inflammatory effect by inhibiting cyclooxygenase [54].

In vitro studies have shown the anti-inflammatory effect of extracts of *Artemisia campestris* by inhibiting lipoxygenase [55]. This species is rich in bioactive compounds such as phenolic acids, flavonoids and terpenoids. Many of the properties of the plant such as antioxidant, antidiabetic, and anti-inflammatory properties are attributed to these compounds.

Another species of *Artemisia*—*Artemisia nilagirica*, which is widely distributed in India—has anti-inflammatory potential, as well as antioxidant, antimicrobial, antifungal, and anticancer properties associated with the presence of phytochemicals such as tannins, flavonoids, alkaloids, saponins, coumarins, steroids and phenols [56,57]. Studies have shown anti-inflammatory properties in other species of the *Artemisia* genus: *Artemisia maritima* L [58] and *Artemisia sieversiana* Ehrh. [59].

## 3. Materials and Methods

### 3.1. Chemicals

All reagents used for the analysis were of analytical purity and were purchased from Sigma Chemical Company (Sigma Aldrich, Darmstadt, Germany), Roth (Carl Roth GmbH, Karlsruhe, Germany), and Fluka (Buchs, Switzerland).

### 3.2. Obtaining the Extracts

The plants, *A. vulgaris* and *A. absinthium*, were harvested in May 2022 from the Orastie area, Hunedoara County, Romania. *T. foenum-graecum* was harvested in April 2021 from Agricola Secuieni Research and Development Station, Neamt County, Romania, where it was cultivated in an ecological farming system. We used seeds from *T. foenum-graecum*; from *Artemisia* sp., we used the aerial part of the plant.

The plants were first dried and finely ground using a GRINDOMIX 200GM mill. Hydroalcoholic extracts of *Artemisia vulgaris* and *Artemisia absinthium* in 50% EtOH (10% mass) were obtained. Extracts were produced via ultrasound-assisted solvent extraction (UAE) at room temperature for 1 h followed by filtration. Ultrasound accelerates heat and mass transfer by destroying plant cell walls, leading to accelerated kinetics and an improved release of bioactive compounds with increased extraction yield.

The extracts were processed by membrane methods: micro- and ultrafiltration via a KMS Laboratory Cell CF-1 laboratory facility purchased from Koch Membrane (Germany).

Microfiltration was performed with microfiltration membranes from regenerated cellulose with a pore size of 0.45 µm for the removal of very fine colloidal particles located between the minimum limit corresponding to conventional filtration (5 µm) and the maximum limit of ultrafiltration (0.1 µm) and re-sterilization, with the result of microfiltration being the microfiltrate. Ultrafiltration was performed with regenerated cellulose membranes with a cut-off of 3000 Da; compounds with a molecular mass lower than the membrane pores pass through the membrane and are found in permeates, while compounds with a molecular mass higher than the membrane pores will be found in concentrates. The microfiltrates and concentrates were analyzed. The concentration was achieved at a ratio of 1:3, and the pressure used was 6 bar.

### 3.3. Bioactive Compounds Determination

The determination of the content of total polyphenols was performed using the slightly modified Folin–Ciocalteu technique [60]. Spectrophotometric measurements were realized at 760 nm. Experiments were carried out in triplicate, and the polyphenol concentration was calculated from an etalon curve of chlorogenic acid (CA).

The total flavonoid content was determined using the colorimetric aluminum chloride assay [61]. The flavonoid content was calculated using the rutin calibration curve and expressed in μg rutin equivalent (RE)/mL of the extract.

HPLC analysis

The chromatographic analysis was performed using a complete HPLC SHIMADZU system and a C18 Nucleosil 3.5, 4.6 × 50 mm, Zorbax column. The system was coupled to an MS detector and an LCMS-2010 detector (liquid chromatograph mass spectrometer), equipped with an ESI interface. The samples were filtrated before injection using Syringe Driven Filter Unit 0.2 µm (Macherey-Nagel).

All the other reagents (acetonitrile, formic acid) were analytical pure or chromatographic grade and were used after filtration. The ultra-pure water was obtained using a system for water purification, Elix 3 (Millipore).

All used standards, chlorogenic acid, gallic acid, ellagic acid, caffeic acid, rosmarinic acid, coumaric acid, rutin, luteolin, quercetin, quercetin 3-β-D-glucoside, apigenin, umbelliferone, kaempferol and genistin stock solutions, 1 mg/mL, were prepared in ethanol. Stock samples were stored in the dark and at −4 °C between experiments.

The HPLC method used for the analysis of polyphenolic compounds was previously published by Alecu et al. [62].

### 3.4. Antioxidant Assays

The antioxidant activity was measured using 3 methods, with ascorbic acid being used as the control in all methods.

1. The method was based on decreasing the maximum absorbance of ABTS to 731 nm in the presence of the antioxidant [63]; antioxidant activity was expressed in TEAC equivalents (Trolox Equivalent Antioxidant Capacity) using the formula
  (1) $${\mathrm{TEAC}}_{\mathrm{sample}}=C_{\mathrm{Trolox}}\cdot f\cdot \frac{A_{\begin{array}{c} \mathrm{sam}\\ \mathrm{ple} \end{array}}-A_{blank}}{A_{Trolox}-A_{blank}}$$
  where A_blank_ = control absorbance; A_Trolox_ = Trolox absorbance. A_sample_ = sample absorbance; f = dilution factor; C_Trolox_ = Trolox concentration.

2. DPPH radical scavenging activity

The scavenging activity on the DPPH radical of samples was determined by measuring the decrease in the DPPH maximum absorbency at 517 nm after 10 min [64] and was calculated as follows:

radical scavenging activity (%) = [(A_B_ − A_A_)/A_B_] × 100 (2)

where: A_B_ = control absorbance and A_A_ = sample absorbance.

○ Reducing Power Activity (Iron (III) to iron (II) reduction)

Reducing power was determined according to a previously described procedure [65]. The absorbance was measured spectrophotometrically at 700 nm and calculation was carried out using the following formula:

Reducing power (%) = [(A_A_ − A_B_)/A_A_] × 100 (3)

where A_A_ = sample absorbance; A_B_ = control absorbance.

### 3.5. Enzyme Inhibitory Activity Assay

#### 3.5.1. Testing the Antidiabetic Capacity of the Extracts

- α Amylase inhibition assay

The α-amylase inhibition assay was performed according to the Ranilla method, which was slightly modified [66]. Thus, 100 μL of extract was mixed with 250 μL of α-amylase from hog pancreas (EC 3.2.1.1) (0.5 mg/mL in 0.02 M sodium phosphate buffer, pH 6.9 with 0.006 M NaCl) and was incubated at 37 °C for 20 min; then, 250 μL of starch solution (1% in sodium phosphate buffer) was added, and the mixture was incubated at 37 °C for 30 min; then, 500 μL of dinitrosalicylic acid (DNS) was added and the mixture was boiled for 5 min. Finally, 5 mL of distilled water was added to the reaction mixture. The absorbance was measured at 540 mm using a UV-visible spectrophotometer (Jasco-V630) with acarbose as the positive control. The calculation of the results was carried out using the following formula:

(4) $$\%\;\mathrm{Amylase}\;\mathrm{inhibition}=\frac{\Delta A_{\mathrm{control}}-\Delta A_{\mathrm{sample}}}{\Delta A_{\mathrm{control}}}\;\times \;100$$

The IC_50_ values were calculated via the linear regression analysis. Significant statistical differences were considered at *p* < 0.05.

- α-Glucosidase inhibition assay

The α-glucosidase inhibition assay was performed according to Queiroz et al. method with slight modification [67]. Thus, 120 μL of α-glucosidase from *Saccharomyces cerevisiae* (EC 3.2.1.20) (0.5 U/mL) was mixed with 720 μL of sodium phosphate buffer (0.1 M, pH 6.9) and 60 μL of extract; then, the mixture was incubated at 37 °C for 15 min. After pre-incubation, 120 μL of p-nitrophenyl–α–D−glucopyranoside (5 mM/L) solution was added, and the reaction mixture was incubated at 37 °C for 15 min. Absorbance was measured at 405 nm using a UV-visible spectrophotometer with acarbose as a positive control. The calculation of the results was based on the following formula:

(5) $$\%\;\mathrm{Glucosidase}\;\mathrm{inhibition}=\frac{\Delta A_{\mathrm{control}}-\Delta A_{\mathrm{sample}}}{\Delta A_{\mathrm{control}}}\;\times \;100$$

The IC_50_ values were calculated via the linear regression analysis. Significant statistical differences were considered at *p* < 0.05.

#### 3.5.2. Testing the Anti-Inflammatory Capacity of the Extracts

○ Hyaluronidase inhibition assay

Hyaluronidase activity (hyaluronidase EC 3.2.1.35 from bovine testis—Sigma) manifested in the hydrolysis of hyaluronic acid, tested using the modified Morgan–Elson method (1949) [68], which involves the measurement at 585 nm of the complex formed by the enzymatic hydrolysis products by a reaction with p-dimethylaminobenzaldehyde.

First, 100 μL of the enzyme solution (1 mg/mL) was pipetted together with 50 μL of the plant extract and the mixture was preincubated at 37 °C for 30 min to inhibit hyaluronic acid center activity. Then, 100 μL of substrate solution (sodium salt of hyaluronic acid in the vitreous bovine humor—2.5 mg/mL) was added to the reaction mixture and incubated at 37 °C for 60 min, the time required to perform the enzymatic reaction. The blank sample was prepared in the same way by replacing the plant extract with a buffer solution.

The reaction was stopped by holding at 100 °C for 3 min, and the reaction products were highlighted by staining with p-dimethylaminobenzaldehyde and the spectrophotometric quantification of the pink complex at 585 nm; the results were calculated according to the following formula:

(6) $$\%\;\mathrm{inhibition}=\frac{\Delta A_{\mathrm{control}}-\Delta A_{\mathrm{sample}}}{\Delta A_{\mathrm{control}}}\;\times \;100$$

The IC_50_ values were calculated via the linear regression analysis. Significant statistical differences were considered at *p* < 0.05.

○ Lipoxygenase inhibition assay

The lipoxidase inhibition assay was performed according to a Sigma-Aldrich protocol [69]: 0.017% (*v*/*v*) linoleic acid substrate and a lipoxygenase solution (2200 units/mL) (EC 1.13.11.12); absorbance was determined to increase for approximately 5 min at 234 nm, using the maximum linear rate for both sample and blank, and results were calculated according to the following formula:

(7) $$\%\;\mathrm{inhibition}=\frac{\Delta A_{\mathrm{control}}-\Delta A_{\mathrm{sample}}}{\Delta A_{\mathrm{control}}}\;\times \;100$$

The IC_50_ values were calculated via the linear regression analysis. Significant statistical differences were considered at *p* < 0.05.

## 4. Conclusions

The phytochemical screening, antidiabetic and anti-inflammatory potential of *Artemisia vulgaris*, *Artemisia absinthium* and *Trigonella foenum-graecum* concentrated hydroalcoholic extracts by inhibiting α-amylase and α-glucosidase and lipoxygenase and hyaluronidase, respectively, were analyzed.

The *T. foenum-graecum* extracts showed higher genistin content—2032.98 μg/mL—and the *Artemisia* species showed higher contents of polyphenols and flavones. The *T. foenum-graecum* extracts showed high antioxidant activity via all methods of analysis: reducing power: 97.11 ± 5.20%; DPPH inhibition: 82.18 ± 3.70%; TEAC_ABTS: 591.23 ± 22.38 mg/mL.

The extracts of *T. foenum-graecum* showed a high inhibitory effect on all the enzymes studied (α-amylase: 3.22 ± 0.30 μg/mL (compared with acarbose standard: 3.50 ± 0.18 μg/mL); α-glucosidase: 11.14 ± 0.90 μg/mL (compared with acarbose standard: 5.90 ± 0.38 μg/mL); hyaluronidase: 17.57 ± 1.23 μg/mL (compared with ibuprofen standard: 5.73 ± 0.21 μg/mL); and lipoxygenase: 19.69 ± 0.52 μg/mL (compared with ibuprofen standard: 20.19 ± 1.25 μg/mL)). The concentrated extract of *Artemisia absinthium* showed high inhibitory activity on LOX: 19.71 ± 0.79 μg/mL (compared with ibuprofen standard: 20.19 ± 1.25 μg/mL), while the concentrated extract of *Artemisia vulgaris* had a significant inhibitory effect on HYA 17.18 ± 1.19 μg/mL (compared with ibuprofen standard: 5.73 ± 0.21 μg/mL). It is noted that the concentrated extracts contained bioactive compounds with significantly higher antidiabetic and anti-inflammatory potential than the initial ones, which shows the efficiency of membrane technologies, specifically ultrafiltration, in the concentration of bioactive compounds.

Thus, these herbs may be potential sources of phytocompounds useful in the treatment of type 2 diabetes and inflammatory diseases. These are preliminary in vitro data that can be supplemented by further studies in appropriate in vivo models.

## Figures and Tables

**Table 1:** Phytochemical analysis and antioxidant capacity of extracts.

Sample		Polyphenol Content (mg CA/mL)	Flavone Content (mg RE/mL)	Reducing Power %	% DPPH Inhibition	TEAC_ABTS mg/mL
*Artemisia absinthium*	MF	3232.5 ± 140.3	389.1 ± 9.5	50.7 ± 1.3	36.1 ± 1.1	204.1 ± 6.3
	concentrate	4777.5 ± 125.5	501.8 ± 26.9	73.5 ± 2.3	65.9 ± 2.3	483.2 ± 3.8
*Artemisia vulgaris*	MF	7877.5 ± 260.9	505.2 ± 30.1	52.9 ± 1.3	63.6 ± 2.5	316.5 ± 9.5
	concentrate	11,440.2 ± 49.9	1020.1 ± 45.1	93.1 ± 3.2	77.5 ± 2.4	541.5 ± 15.3
*Trigonella foenum-graecum*	MF	1094.3 ± 62.4	395.8 ± 11.6	63.8 ± 2.4	75.4 ± 5.8	420.7 ± 17.3
	concentrate	2636.8 ± 52.7	777.6 ± 2.7	97.1 ± 5.2	82.2 ± 3.7	591.2 ± 22.3
Ascorbic acid				34.5 ± 1.3	96.9 ± 3.6	

Values represent the mean standard deviation of triplicate experiments.

**Table 2:** Polyphenolic compound contents of the extracts—analysis via HPLC-PDA-MS.

Compound	*A. absinthium* MF µg/mL	*A. absinthium* Concentrate µg/mL	*A. vulgaris* MF µg/mL	*A. vulgaris* Concentrate µg/mL	*T. foenum-graecum* MF µg/mL	*T. foenum-graecum* Concentrate µg/mL
Chlorogenic acid	259.4	297.9	301.1	350.6	11.1	68.1
Caffeic acid	2.6	3.2	2.4	3.1	-	-
Rosmarinic acid	21.0	22.6	3.3	3.1	-	-
Coumaric acid	-	-	-	-	7.3	7.7
Umbelifferone	6.9	7.9	7.1	8.9	-	-
Quercetol	0.6	0.7	0.8	0.1	-	-
Luteolin	4.7	5.5	12.4	14.5	3.5	3.9
Apigenin	0.7	1.1	2.5	2.6	-	-
Rutin	12.6	15.6	18.4	19.6	u.d.l	84.9
Ellagic acid	4.7	5.1	-	-	-	-
Isoquercitrin	1.5	1.9	3.7	3.7	7.3	20.1
Genistin	-	-	-	-	1286.2	2032.9

u.d.l.—under the detection limit.

**Table 3:** In vitro inhibition of α-amylase and α-glucosidase activity of extracts.

Samples		Inhibition of α-Amylase IC_50_ (μg/mL)	Inhibition of α-Glucosidase IC_50_ (μg/mL)
*A. absinthium* extracts	MF	22.2 ± 0.9	45.16 ± 1.4
	concentrate	19.4 ± 0.5	31.90 ± 1.8
*A. vulgaris* extracts	MF	17.0 ± 0.9	96.04 ± 3.2
	concentrate	8.5 ± 2.3	77.13 ± 2.3
*T. foenum-graecum* extracts	MF	24.1 ± 1.4	28.19 ± 1.8
	concentrate	3.2 ± 0.3	11.14 ± 0.9
Rosmarinic acid		0.9 ± 0.1	0.18 ± 0.1
Chlorogenic acid		1.9 ± 0.1	0.59 ± 0.2
Acarbose		3.5 ± 0.1	5.90 ± 0.3

Values represent the mean standard deviation of triplicate experiments.

**Table 4:** In vitro inhibition of HYA and LOX activity of extracts.

Samples		Inhibition of HYA IC_50_ (μg/mL)	Inhibition of LOX IC_50_ (μg/mL)
*Artemisia absinthium* extracts	MF	78.1 ± 2.3	52.9 ± 2.1
	concentrate	34.7 ± 1.2	19.7 ± 0.8
*Artemisia vulgaris* extracts	MF	74.1 ± 2.6	182.4 ± 10.5
	concentrate	17.2 ± 1.2	112.7 ± 8.5
*T. foenum-graecum* extracts	MF	67.4 ± 2.6	31.1 ± 1.3
	concentrate	17.6 ± 1.2	19.7 ± 0.5
Ibuprofen		5.7 ± 0.2	20.2 ± 1.2
Rosmarinic acid		-	30.3 ± 1.2
Chlorogenic acid		-	26.1 ± 1.3
Rutin		-	22.34 ± 1.9

Values represent the mean standard deviation of triplicate experiments.

## Data Availability

The authors confirm data supporting the findings of this study available within the article.
